# The toxicokinetics cell demography model to explain metal kinetics in terrestrial invertebrates

**DOI:** 10.1007/s10646-012-0972-6

**Published:** 2012-07-10

**Authors:** Krzysztof Argasinski, Agnieszka Bednarska, Ryszard Laskowski

**Affiliations:** Institute of Environmental Sciences, Jagiellonian University, Gronostajowa 7, 30-387 Kraków, Poland

**Keywords:** Toxicokinetics, Metals, Toxic chemicals, Model, Population, Cell replacement, Physiological mechanism, Cell demography

## Abstract

Metal toxicokinetics in invertebrates are usually described by one-compartment first-order kinetic model. Although the model gives an adequate description of the toxicokinetics in certain cases, it has been shown to fail in some situations. It also does not seem acceptable on purely theoretical grounds as accumulation and excretion rates may change depending on instantaneous toxicant concentration in the gut. We postulate that the mechanism behind such changes is connected with the toxic effect of metals on gut epithelial cells. Based on published data, we have constructed a mechanistic model assuming a dynamic rate of replacement of epithelial cells with increasing contamination. We use a population-type modeling, with a population of gut epithelial cells characterized by specific death and birth rates, which may change depending on the metal concentration in food. The model shows that the equilibrium concentration of a toxicant in an organism is the net result of gut cell death and replacement rates. At low constant toxicant concentrations in food, the model predicts that toxicant-driven cell mortality is moderate and the total amount of toxicant in the intestine increases slowly up to the level resulting from the gradual increase of the cell replacement rate. At high constant concentration, total toxicant amount in the gut increases very fast, what is accompanied by massive cell death. The increased cell death rate results in reduced toxicant absorption, which in turn brings its body load down. The resulting pattern of toxicokinetic trajectory for high metal concentration closely resemble that found in empirical studies, indicating that the model probably describes the actual phenomenon.

## Introduction

### Classic toxicokinetic model—critique

Toxicokinetics (TK) was an important area of research well before ecotoxicology has been invented. Understanding the kinetics of a toxic chemical is the very basis for studies on its distribution and toxicity in an organism. Not surprisingly, specific TK models have been developed in pharmacology and medicine to describe and predict the behavior and fate of toxic chemicals (and drugs alike) in animals. Physiology-based pharmacokinetic (PBPK) models can be fairly complicated; they incorporate a number of different body parts (“compartments”: the intestine, liver, kidneys, etc.) and processes (absorption, distribution, metabolism, excretion). In ecotoxicology, and especially in the case of metal TK, metabolism is usually neglected (metals cannot be degraded like pesticides), and only absorption and excretion rates are studied. This simplifies the model greatly, still allowing the internal concentrations of metals to be predicted in animals inhabiting metal-polluted environments.

The TK of metals traditionally have been described by a simple one-compartment two-phase model which assumes that the net metal accumulation rate—and hence its final body concentration—depends on the balance between the metal absorption rate *k*_*a*_ and excretion rate *k*_*e*_. The process can be described as a system of linear differential equations. In the classic one-compartment toxicokinetic model (Atkins 1969) the dynamic of internal concentration of a toxic chemical, ${\dot{\text{C}}}_{\text{int}}$, which is absorbed at rate *k*_*a*_ from the external environment (e.g., food), contaminated at concentration *C*_ext_, and eliminated from the organism at rate *k*_*e*_, is described by the equation:

$$ \dot{C}_{\text{int}} = k_{a} C_{\text{ext}} - k_{e} C_{\text{int}} $$

Although in some cases such a model describes metal kinetics satisfactorily, it is actually not a proper description of the underlying mechanisms but only a good approximation as the approach is purely phenomenological. The classic one-compartment model allows only for an asymptotic approach to a stable concentration resulting directly from the balance between *k*_*a*_ and *k*_*e*_, which are constant throughout the exposure period. Although broadly accepted and used in TK modeling, such an approach seems neither confirmed by data (Janssen et al. 1991; Lagisz et al. 2005) nor reasonable from the biological point of view. Moreover, the model relies on the assumption of linearity of absorption and excretion processes but our recent studies indicated that under certain circumstances the two-phase model does not fit the trajectory of metal concentrations in animals exposed to metal-contaminated food (Bednarska et al. 2011; Laskowski et al. 2010). A number of other studies also showed similar deviations from the classical two-phase model (Descamps et al. 1996; Lagisz et al. 2005; Janssen et al. 1991).

A change in the physiology of metal regulation as an outcome of poisoning of gut epithelial cells and replacement of dead cells with new ones is one of the mechanisms we suggested to explain nickel TK in our previous work on nickel TK in the ground beetle *Pterostichus oblongopunctatus* (Bednarska et al. 2011). We used these data herein to show that the model can indeed describe a real-world situation. These data spurred us to rethink the whole framework of metal TK—largely due to the unexpected Ni TK, which could not be explained by the classic one-compartment two-phase model. Later we found a number of studies highlighting similar problems (Laskowski et al. 2010). Briefly, we observed that sometimes the body metal concentrations began to decrease when an animal was still exposed to metal-contaminated food. During exposure to contaminated food, the initial quick increase in body metal concentration (phase I) is followed by an asymptotic decrease to a certain equilibrium level (phase II) higher than the initial concentration. If an animal is then switched to uncontaminated food, the concentration drops once again (phase III) and, depending on excretion efficiency, can ultimately reach the pre-exposure concentration (Laskowski et al. 2010). Approximating such kinetics requires at least three or four different constants instead of two, as it assumes one of the following:

1. There is one *k*_*a*_ and two elimination constants: one for phase I (*k*_*e1*_) and a different one for phase II (*k*_*e2*_), such that *k*_*e1*_ < *k*_*e2*_. Then the initial fast increase would result from low *k*_*e1*_ during the first period of exposure, and after a certain time the elimination rate increases to *k*_*e2*_, resulting in a concentration decrease; *k*_*e2*_ operates also when the animal switches to uncontaminated food, allowing for (almost) complete excretion of the toxicant.
2. There are two absorption constants, *k*_*a1*_ > *k*_*a2*_, and one *k*_*e*_. At the start of exposure the high *k*_*a1*_ dominates over *k*_*e*_, resulting in a fast increase of metal concentration in the body. At a certain concentration (or after some time of intoxication) the absorption rate decreases to *k*_*a2*_, leading to a concentration decrease to a new equilibrium level. Again, if an animal is transferred to uncontaminated food, the concentration of a metal in its body can decrease to the pre-exposure level, as there is no further absorption while excretion is still active.
3. The third scenario is a combination of (1) and (2), allowing for two absorption constants and two elimination constants. The observed result would be the same as in (1) and (2).

To accommodate that unexpected pattern Laskowski et al. (2010), proposed a three-phase model. The proposed solution was purely phenomenological, however. Formulating a coherent mechanistic TK model based on animal physiology was the main goal of the work we describe here.

### The physiology of metal regulation in invertebrates

Only carefully designed studies incorporating the TK of several metals and the dynamics of epithelial cell populations exposed to these metals can truly confirm the model’s assumption of the gut epithelial cell death rate as the main mechanism determining metal absorption and excretion rates. Such data do not exist at the moment. It is known, however, that insects can protect themselves against harmful effects of metals by storing them in intra-cellular crystalline structures (granules). After cell death and degradation, metals accumulated in these structures are released into the gut lumen and gradually eliminated from the organism (Hopkin 1989; Rost-Roszkowska et al. 2008). Insect midgut epithelial cells, which are responsible for digestion, absorption and secretion, die in one of the two processes: necrosis and apoptosis. Both processes can participate in the removal of excessive metals from the gut. The discharged degenerated cells are replaced by new ones, differentiated from the regenerative cells (Rost-Roszkowska et al. 2009). The increased removal of gut epithelial cells contaminated with metals has been hypothesized to be the mechanism responsible for an increased metal excretion efficiency in the metal exposed beetle *Pterostichus niger* (Lindqvist et al. 1995). Also Migula et al. (2011) proved that apart from feeding behaviour, the fast replacement of midgut epithelial cells overloaded with Ni is the main protective mechanisms against Ni toxicity in the beetle *Epilachna nylanderi*.

### The new framework

Organisms are known for the flexibility of their responses to environmental conditions. They employ a myriad of physiological and biochemical compensatory mechanisms to maintain reasonably stable internal conditions over a range of variation of external conditions. For example, a homeothermic organism exposed to low temperature generates heat by increasing its metabolic rate, and does so proportionally to the needs—there is no point in maximizing the metabolic rate if the ambient temperature is only a little below optimum. The metabolic rate is thus regulated by body requirements for heat, ultimately sensed by body surface temperature. In contrast, the classic two-phase TK model allows *k*_*a*_ and *k*_*e*_ to change only with the external metal concentration, without reference to the internal body metal concentration. This seems wrong from a purely physiological point of view: it is the internal concentration that should be regulated and which should be monitored by an organism. Nor does it make sense in evolutionary terms: optimal allocation of resources, promoted by natural selection, should allow the energy expenditure on absorption and excretion to be regulated proportionally to instantaneous needs. Thus, from a biological perspective it is a more plausible scenario for absorption and excretion rates to change dynamically depending on the current status of an organism (i.e., internal metal concentration).

The patterns of metal uptake and excretion in invertebrates differ vastly, both between species and between metals. For example, ground beetles are relatively efficient in maintaining constant metal concentrations in their bodies (Heikens et al. 2011; Janssen et al. 1991; Kramarz 1999a), while arachnids are not able to control body metal concentrations efficiently and accumulate metals in extremely high concentrations when overexposed to them in food (Heikens et al. 2011; Janssen et al. 1991; Wilczek et al. 2004). Within species, substantial differences between metals are seen. The zinc concentration usually is relatively well regulated: almost perfectly in carabids (Kramarz 1999a) and less so in centipedes (Kramarz 1999b). Apparently it is more difficult to regulate the cadmium concentration, as even in ground beetles its level rises under increased ingestion (Janssen et al. 1991; Kramarz 1999a). These differences suggest that there are major physiological limitations which prevent organisms from achieving similarly efficient metal regulation across species, or that different strategies are optimal for different organisms and metals. These two explanations are not mutually exclusive, and both find support in literature data (Heikens et al. 2011; Lindqvist et al. 1995); those researchers drew different conclusions from their work, but that is due at least in part to differences in their experimental setups and to their adherence to the classical two-phase model.

Here we propose a completely different approach which we call TK cell demography model (TKCD). In this model the metals themselves affect absorption and excretion by damaging gut epithelial cells, the first line of defense against excessive levels of metals (Rost-Roszkowska et al. 2008; Vandenbulcke et al. 1998). Within this approach there is no need to recognize separate phases or define phase-specific absorption and excretion rates, because both processes are the direct outcome of instantaneous metal concentrations and their effects in cells. Briefly, the higher the concentration and toxicity of a metal, the higher the cell mortality and the lower the absorption rate. Moreover, because absorption and excretion rates result directly from instantaneous toxicity, they may change dynamically depending on the current metal concentration in the gut epithelium.

The main goal of our modeling approach was to create a minimal set of assumptions allowing us to generate patterns observed in previous studies (Bednarska et al. 2011; Descamps et al. 1996; Laskowski et al. 2010), so the model is extremely simplified. An individual is reduced to an intestine consisting of cells of the same type. An individual cell can only absorb toxic particles and die with some probability according to the intrinsic concentration, and there are no cell repair or cell decontamination mechanisms.

The mathematical framework applied in this model is similar to that of demographic models. The methodology can thus be called cell demography. Each cell and each toxicant particle (e.g., metal ion) is treated as a single individual. As an effect of the interaction between a cell and a toxicant, the cell either moves to another contamination class or dies with a certain probability. For technical reasons, in our model the cells are divided into separate contamination classes, which differ in rates of mortality caused by absorbed toxicants. This paper describes a model of a single organ, the intestine, but the methodology can also treat a whole organism as a colony of unicellular organisms. In that case, other methods from population dynamics or evolutionary game theory can be used to describe processes within an organism: cells are individuals and different types of cells result from the division of labor within the colony.

## Methods

The model allows for interspecific differences resulting from (a) differences in the sensitivity of epithelial cells to a metal, (b) possible differences in the rates of turnover (proliferation) of epithelial cells, and (c) differences in exposure. Metals (or any toxic chemicals for that matter) differ in their inherent toxicity. For example, cadmium usually is more toxic than zinc. Their concentrations in food available in nature also differ. The ultimate toxic effect is thus the product of the metal’s inherent toxicity and concentration. The effects of metals differing in their toxicity to gut epithelial cells can be incorporated into the model in three alternative ways, depending on the shape of the relationship between the concentration of each metal and its toxic effects:

1. If the relationships have different shapes for the metals considered, then both the number of metal ions entering cells and the metal toxicities have to be specified explicitly. In the model description (see below), the former is specified as the value of parameter Δ_C_(*t*), and the latter by cell mortality, which is a function of the toxic chemical concentration in the cell. This is a continuous function whose shape is determined by some phenomenological parameters. To apply differential equations, however, the contamination classes must be discrete and limited to a finite number. The evolution of each contamination class is described by a separate equation. Contamination class-specific mortalities (*d*_*i*_) are values of the general mortality function.
2. On the other hand, if we assume the same shape of the concentration-death rate function and an additive toxicity model for the metals, parameter Δ_C_(*t*) can be understood as the change in “effective concentration”, expressed in toxic units (see, e.g., De March and De March (1987) for toxic unit), while keeping the same *d*_*i*_ values for different metals (or any other toxicant fulfilling the above criteria).
3. The third case is a combination of the first two approaches. The metals are characterized by effective toxicity as in case (2), but the effective toxicity is defined as the amount necessary to obtain maximum mortality (to kill all cells), not the amount normalized to every contamination class as it is defined in case (2). Then, in each toxicity class the substances may differ in the shape of the mortality function as in case (1).

Thus, in case (1) the model describes the effects and TK of just one toxicant. For any other toxicant with a different shape of the concentration-effect relationship the death rates of cell contamination classes would need to be adjusted to represent its actual toxicity. In the simpler case (2), the model can be assumed to describe the effects and kinetics of a range of chemical substances whose toxicity to gut cells can be described by an additive model and the concentrations are expressed in toxic units. In the more detailed case (3), substances differ in toxic strength and the intensity of cell-killing, characterized by the shape of the mortality function. In each case the model operates on discrete contamination classes described by a differential equation. The number of contamination classes can be interpreted as the “resolution” of the model.

Numerical trajectories are produced with a constant cell birth rate. This means that there is no organism response to damage, which should be expected in some form in living organisms. In general the cell birth rate may be a function of, for example, the state of the intestine, but we need data to justify this assumption and to estimate the shape of this function. Our model is simplified according to the current state of knowledge. However, even such a simple model can produce fairly complex dynamics similar to the patterns observed in reality and shows the direction of future empirical research in the field.

To avoid misunderstandings we should clarify that the model does not deal with mortality of the whole organism, which is reduced to the intestine, but it shows damage to the intestine caused by toxic chemicals. The relationship between the scale of damage and individual mortality is not a subject of this paper, but further investigations on this relationship should be a natural consequence of the results presented here. The model describes the dynamics of changes in the number and contamination of intestine cells corresponding to the absorption of a toxicant by an organism.

As in our model we focus on the intestine, in this case *C*_int_ would mean the amount of toxic chemical aggregated in the intestine rather than the whole organism (although it can be equivalent to the whole-organism concentration if the toxic chemical does cross the intestine barrier). Below is a detailed description of the model proposed in this work. The following notation is used throughout the model description: *b*—number of new cells produced during $\Updelta t;$ for simplicity, basically we assume that this parameter is constant. Considering the organism’s response to damage, however, the value of this parameter could be a function of, for example, intestine size (damage may induce increased production of intestine cells). *l* number of contamination classes, Δ_*C*_(*t*) number of toxic particles introduced to organism during interval Δ*t*, *N*_*i*_ number of cells in *i*-th contamination class with cell concentration *i*, $N\left( t \right) = \sum\nolimits_{i = 1}^{l} {N_{i} \left( t \right)}$ size of intestine, *d*_*i*_ probability of death of the cell in *i*-th contamination class (*d*_*i*_ < *d*_*i*+1_ and *d*_1_ > 0 and *d*_*l*_ = 1).

Assume that intestine consist of cells that can absorb toxic particles. The number of toxic particles (or toxic units) is smaller than number of cells ($\Updelta_{c} \left( t \right) < N\left( t \right)$). Then, in each short time interval $\Updelta t$, *b* new uncontaminated cells are produced and each cell currently present in the intestine can die with probability $d_{i}$ determined by the current concentration of toxic particles in the cell or else not die with probability $\left( {1 - d_{1} } \right)$ and then absorb toxic particle (or toxic unit) with probability $\frac{{\Updelta_{c} \left( t \right)}}{N\left( t \right)}$. After absorption of a toxic particle, the cell changes its contamination class. Assume that rates are proportional to probabilities described above. From formal point of view, all right sides of above equations should be multiplied by some time scale constant (or reaction rate constant) equal $1/\Updelta t$. However, for simplicity, rate constant can be neglected, since it does not affect qualitative properties of the system. According to these assumptions, system of ordinary differential equations describing evolution of the state of organism can be formulated:

$$ \dot{N}_{1} \left( t \right) = b - d_{1} N_{1} \left( t \right) - \left( {1 - d_{1} } \right)\frac{{\Updelta_{c} \left( t \right)}}{N\left( t \right)}N_{1} \left( t \right) $$

$$ \dot{N}_{2} \left( t \right) = \left( {1 - d_{1} } \right)\frac{{\Updelta_{c} \left( t \right)}}{N\left( t \right)}N_{1} \left( t \right) - d_{2} N_{2} \left( t \right) - \left( {1 - d_{2} } \right)\frac{{\Updelta_{c} \left( t \right)}}{N\left( t \right)}N_{2} \left( t \right) $$…

$$ \dot{N}_{i} \left( t \right) = \left( {1 - d_{i - 1} } \right)\frac{{\Updelta_{c} \left( t \right)}}{N\left( t \right)}N_{i - 1} \left( t \right) - d_{i} N_{i} \left( t \right) - \left( {1 - d_{i} } \right)\frac{{\Updelta_{c} \left( t \right)}}{N\left( t \right)}N_{i} \left( t \right) $$…

$$ \dot{N}_{l} \left( t \right) = \left( {1 - d_{l - 1} } \right)\frac{{\Updelta_{c} \left( t \right)}}{N\left( t \right)}N_{l - 1} \left( t \right) - N_{l} \left( t \right) $$

after simplification:

$$ \dot{N}_{1} \left( t \right) = b - \left( {d_{1} + \left( {1 - d_{1} } \right)\frac{{\Updelta_{c} \left( t \right)}}{N\left( t \right)}} \right)N_{1} \left( t \right) $$

$$ \dot{N}_{2} \left( t \right) = \left( {1 - d_{1} } \right)\frac{{\Updelta_{c} \left( t \right)}}{N\left( t \right)}N_{1} \left( t \right) - \left( {d_{2} + \left( {1 - d_{2} } \right)\frac{{\Updelta_{c} \left( t \right)}}{N\left( t \right)}} \right)N_{2} \left( t \right) $$…

$$ \dot{N}_{i} \left( t \right) = \left( {1 - d_{i - 1} } \right)\frac{{\Updelta_{c} \left( t \right)}}{N\left( t \right)}N_{i - 1} \left( t \right) - \left( {d_{i} + \left( {1 - d_{i} } \right)\frac{{\Updelta_{c} \left( t \right)}}{N\left( t \right)}} \right)N_{i} \left( t \right) $$…

$$ \dot{N}_{l} \left( t \right) = \left( {1 - d_{l - 1} } \right)\frac{{\Updelta_{c} \left( t \right)}}{N\left( t \right)}N_{l - 1} \left( t \right) - N_{l} \left( t \right) $$

In case of a healthy not contaminated organism ($\Updelta_{c} \left( t \right) = 0$) we have:

$$ \dot{N}_{1} \left( t \right) = b - d_{1} N_{1} \left( t \right) $$

and in effect we obtain the initial conditions for the contamination model:

$$ N_{1} \left( 0 \right) = \frac{b}{{d_{1} }} $$

The amount of toxic particles aggregated in the organism is

$$ C_{\text{int}} = \sum\nolimits_{i = 1}^{l} {N_{1} \left( {i - 1} \right)} $$

Because the system is nonlinear, it is hard to calculate stationary points. It is possible to calculate the proportionality relationships between contamination classes dependent on intestine size, under assumption that parameter Δ_*c*_ is constant:

$$ N_{1} = \frac{b}{{d_{1} + \left( {1 - d_{1} } \right)\frac{{\Updelta_{c} }}{N}}} $$

$$ N_{2} = \frac{{\left( {1 - d_{1} } \right)\frac{{\Updelta_{c} }}{N}}}{{d_{2} + \left( {1 - d_{2} } \right)\frac{{\Updelta_{c} }}{N}}}N_{1} = \frac{{\left( {1 - d_{1} } \right)\frac{{\Updelta_{c} }}{N}b}}{{\left( {d_{2} + \left( {1 - d_{2} } \right)\frac{{\Updelta_{c} }}{N}} \right) \left( {d_{1} + \left( {1 - d_{1} } \right)\frac{{\Updelta_{c} }}{N}} \right)}} $$…

$$ N_{i} = \frac{{\left( {1 - d_{i - 1} } \right)\frac{{\Updelta_{c} }}{N}}}{{d_{i} + \left( {1 - d_{i} } \right)\frac{{\Updelta_{c}}}{N}}}N_{i - 1} = \frac{{b\prod\limits_{f = 1}^{i - 1} {\left( \left( {1 - d_{f - 1} } \right)\frac{{\Updelta_{c}}}{N}\right)} }}{{\prod\limits_{f = 1}^{i} {\left( {d_{f} + \left( {1 - d_{f} } \right)\frac{{\Updelta_{c}}}{N}} \right)} }} $$…

$$ N_{1} = \left( {1 - d_{l - 1} } \right)\frac{{\Updelta_{c} }}{N}N_{l - 1} = \frac{{b\prod\limits_{f = 1}^{l - 1} {\left( {1 - d_{f - 1} } \right)\frac{{\Updelta_{c} }}{N}} }}{{\prod\limits_{f = 1}^{l} {\left( {d_{f} + \left( {1 - d_{f} } \right)\frac{{\Updelta_{c} }}{N}} \right)} }} $$

The case for five contamination classes was run with SciLab ver. 5.2.1 (Scilab Consortium/Digiteo; http://www.scilab.org).

Cell death probabilities were chosen arbitrarily and equal to *d*_1_ = 0.1, *d*_2_ = 0.11, *d*_3_ = 0.2, *d*_4_ = 0.9, and *d*_5_ = 1, birth rate *b* = 10. Each simulation was started from the initial condition *N*_1_(0) = *b*/*d*_1_ = 100 describing equilibrium of cellular turnover in uncontaminated organism. The total number of cells at *t*_0_ was scaled to 100 % so that the model was not restricted to any specific number of epithelial cells, and the model output can be understood in terms of percentage damage of the gut epithelium. Because the model can accept any initial input concentration/toxicity, the cell death rate can become so high in certain scenarios that most cells die soon after an animal is exposed to a toxicant. Therefore, biologically sensible are the trajectories where the condition $\frac{{\Updelta_{c} \left( t \right)}}{N\left( t \right)}$ ≤ 1 is satisfied because absorption probability cannot exceed one.

## Results and discussion

**Fig. 1:**
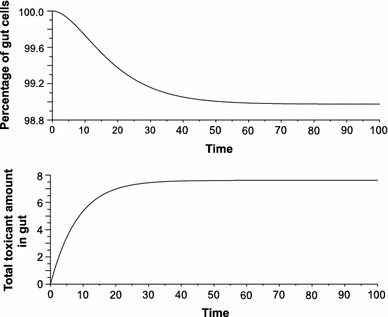
Model predictions for low toxicity scenario ($\Updelta_{C} (t)$ = 1, *b* = 10 show only minor cell losses (*upper panel*) and low amount of the toxicant (nominal value 7.5) accumulated in gut epithelial cells (*lower panel*); $\Updelta_{C} (t)$ number of toxic particles introduced to the organism during interval $\Updelta t$, *b* number of new cells produced during $\Updelta t$

**Fig. 2:**
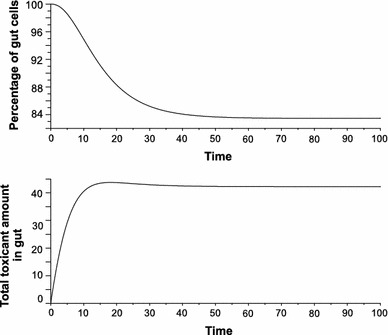
Model predictions for moderate toxicity ($\Updelta_{C} (t)$ = 9, *b* = 10) show ca. 16 % loss of cells (*upper panel*) and a marked increase in the amount of the toxicant (nominal value 45) accumulated in gut epithelial cells, with only a slight decrease after ca. 15 time units (*lower panel*); $\Updelta_{C} (t)$ number of toxic particles introduced to the organism during interval $\Updelta t$, *b* number of new cells produced during $\Updelta t$

**Fig. 3:**
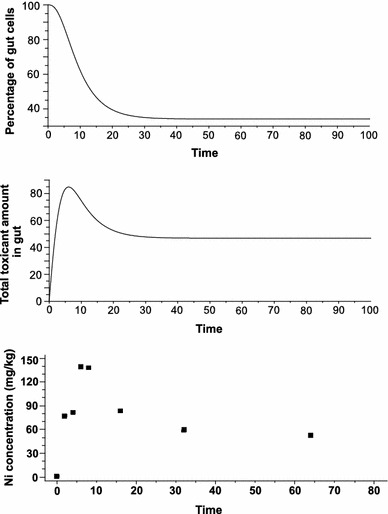
Model prediction for high toxicity ($\Updelta_{C} (t)$ = 34, *b* = 10) show massive loss of cells (ca. 65 %; *upper panel*) and a clear peak in toxicant content in gut epithelial cells (*middle panel*). After reaching the maximum amount in gut epithelial cells, the amount of toxicant decreases to the level observed in the scenario presented on Fig. 2; This trajectory is similar to the pattern observed in an earlier empirical study (*lower panel*): actual whole-body Ni concentrations in *Pterostichus oblongopunctatus* exposed to Ni-contaminated food (Bednarska et al. 2011). Please note that the TKCD-generated curve has not been fitted to the data but rather generated with the assumed parameters as described for Fig. 3 (see also “Methods” section)

The model’s performance was tested using a range of constant concentration/toxicity values (Figs. 1, 2, 3). So far the model has been tested whether it can follow real experimental data on metal TK. Since the TKCD model is mechanistic rather than phenomenological or statistical regression model, fitting its parameters, such as cell mortalities, to the observed data is not methodologically correct. Technically this is possible but the estimated parameter values would be not scientifically plausible. The TKCD model predictions for high toxicity were compared with results of the empirical study on *Pterostichus oblongopunctatus* exposed to 2,500 mg Ni/kg dry food (Bednarska et al. 2011)—the only Ni concentration used by the authors. Figure 3 shows that the model produces pattern similar to the empirical observations despite its simplicity. Accordingly, laboratory studies gathering TK of different metals at different concentrations in food would be invaluable in testing the model’s performance. As stated by Forbes et al. (2009), models can identify important data gaps which can be used to guide further study designs—and that is exactly what results from the model presented herein.

### Classical theory from the TKCD point of view

Classical multicompartment models (Godfrey 1983) rely on the phenomenological assumption that transport from one compartment to another can be described by linear transfer constant *k*. The presented model assumes that the incidental transfer rate depends on the instantaneous concentration of a toxicant and is determined by the instantaneous state of the intestine. From the classical theory point of view, the crucial question is how the TKCD model affects the assumption of linearity of the decontamination process. The cell demographic model shows that the amount of toxic particles removed from the intestine during short time interval $\Updelta t$ is equal to $\sum\nolimits_{i = 1}^{l} {N_{i} d_{i} \left( {i - 1} \right)}$ (sum of all cells removed from each contamination class multiplied by the amount of contained toxic particles). Dividing this value by the intestinal concentration of the toxic chemical results in an instantaneous local value of secretion rate which is analogous to elimination rate from classical theory:

$$ k\left( {N,N_{1} , \ldots ,N_{l} } \right) = \frac{{\sum\nolimits_{i = 1}^{l} {N_{i} d_{i} \left( {i - 1} \right)} }}{{\sum\nolimits_{i = 1}^{l} {N_{i} \left( {i - 1} \right)} }} = \frac{{\sum\nolimits_{i = 1}^{l} {q_{i} d_{i} \left( {i - 1} \right)} }}{{\sum\nolimits_{i = 1}^{l} {q_{i} \left( {i - 1} \right)} }}\quad {\text{where}}\:\;q_{i} = \frac{{N_{i} }}{N} $$

**Fig. 4:**
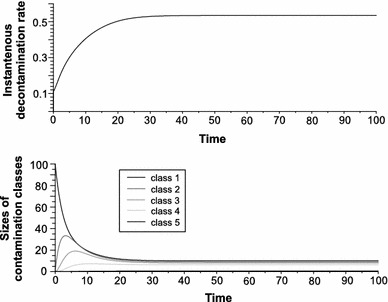
Trajectory of instantaneous elimination rate (*upper panel*) and underlying evolution of intestine state as described by changes of contamination class sizes (*lower panel*, class 1 described healthy cells and next classes are more contaminated). Plotted for conditions from Fig. 3

Therefore it is not a constant but a nonlinear function independent of intestine size, but it depends on the current state of the intestine as described by the relative sizes of contamination classes *q*_*i*_. Figure 4 shows the trajectory of the elimination rate and the underlying evolution of the intestine’s state (changes of sizes of contamination classes). The trajectory of the elimination rate starts at 0.11, which is the death rate in the second contamination class (the lowest class where toxins are present in the organism), and increases until the state of the intestine reaches equilibrium. Now it is clear that classical theory can be regarded as the case of a single contamination class. In this case, however, there is no neutral cell mortality and in effect no cell turnover (in the TKCD model the death probability in the last contamination class is equal to one to induce turnover). Cells in living organisms are complex objects with specific life cycles and cannot be characterized by a half-life as radioactive particles are in isotopic tracer kinetics. The TKCD model makes it clear that the assumption of linearity of transport is not defensible.

The new model generalizes the multicompartment approach to each cell: every cell is a separate compartment, and the number of compartments may change during the contamination period. In standard multicompartment models the different compartments refer to different organs/tissues. Equivalent to this approach, a cell demographic model should consider several cell populations of tissues from different organs and describe the relationships between them (mechanisms of transport of toxic chemicals from one organ to another). This is one possible way to further develop and generalize cell demographic modeling in (eco)toxicology.

The TKCD model can be verified by measuring the parameters and initial conditions empirically and then comparing the trajectories with observations. The reality will probably prove to be more complex than the reasoning we have followed here, and empirical studies will turn up a plethora of factors affecting this process. At that point the model can be fleshed out and made more realistic. The detailed studies needed to verify and improve the model are the next step.

### Cell demography framework and biology

The perspective presented in this work can be of value for modeling evolutionary processes because it may provide the link between life history optimization (Roff 1992; Stearns 1992) and dynamic energy budget (DEB) theory (Kooijman 2000). The two theories operate at different levels. Life history theory investigates, for example, how natural selection shapes allocation of energy to growth or reproduction. In this approach the organism is treated as a black box implementing decisions about resource allocation, which is optimized under certain conditions by the action of evolution (selection). The predictions of this theory may be physiologically unrealistic. On the other hand, DEB theory treats the organism as a complex chemical process. Here physiological realism is highly emphasized, but the lack of an evolutionary perspective is a problem. For example, in DEB models it is assumed that an organism first invests in repair and after that in reproduction. This contradicts some predictions of life history theory (Roff 1992; Stearns 1992). DEB theory uses the classical multicompartment framework to describe physiological processes. Applying cell population dynamics will allow DEB theory to be linked with the life history perspective. Physiological reactions are realized at the level of a single cell, and natural selection affects allocation of resources to production of different types of cells to optimize the work of the whole system (cell colony).

Currently, the empirical research methodology in ecology does not deal with processes operating on cellular level. Our model suggests that physiological mechanisms driven by cells activity play crucial role and should be a major subject of experimental work. We believe that the model already provides an interesting alternative to the classic toxicokinetic models and we hope that researchers will thoroughly test it against laboratory observations in coming years. Toxicokinetics cell demography makes up a new research framework which can be of use in addressing a range of problems wider than ecotoxicology. In the more general perspective, cell demography bears upon several controversial concepts discussed in modern biology. It is related to the field of systems biology, describing the relationships between processes operating in living organisms and on higher levels of organization. For example, the whole symbiogenesis concept (Kozo-Polyansky 2010) is based on an approach similar to cell demography. It assumes that unicellular organisms develop symbiotic relationships and in effect join together into a multicellular organism as a system. From an evolutionary point of view, cell population modeling is a generalization of the concept of multilevel selection (Wilson et al. 2008), with the assumption that every multicellular organism is a colony of unicellular organisms which are closely related and have highly organized relationships between them. Similar way of thinking about organism as an ecosystem was applied in recent works on cancer modeling (Kareva 2011; Merlo et al. 2006; Nowell 1976). Also Brown et al. (2006) applied such a demographic approach to *Salmonella enterica* infection; they considered demographic structures of bacterial populations colonizing cells. Following that reasoning, every group of multicellular organisms is a higher-order structure—a colony of colonies. In the biology of social insects there is an influential concept of the “superorganism” (Hölldobler and Wilson 2008), describing a highly organized colony of relatively simple organisms such as ants as a kind of an individual organism. Our approach goes exactly opposite way, applying population thinking to single organ of a multicellular individual. Our understanding of the organism as an organized population of cells is nothing unusual.
